# Longitudinal Study on Dental Status, Caries Risk Assessment, and Oral Health-Related Quality of Life in 3-6-Year-Old Children Receiving Nitrous Oxide Inhalational Sedation

**DOI:** 10.7759/cureus.59051

**Published:** 2024-04-26

**Authors:** Nandini Devi M, Vignesh Ravindran

**Affiliations:** 1 Department of Pedodontics and Preventive Dentistry, Saveetha Dental College and Hospitals, Saveetha Institute of Medical and Technical Sciences (SIMATS), Saveetha University, Chennai, IND

**Keywords:** dental status, caries risk, oral health, quality of life (qol), nitrous oxide oxygen sedation

## Abstract

Introduction

Dental caries prevention in early childhood is crucial, necessitating timely assessments by dental professionals and parents. The oral health of children during their early years can significantly impact both the children themselves and their parents' quality of life. This study seeks to assess the changes in oral health-related quality of life after treatment under nitrous oxide sedation in anxious preschool children, for whom treatment otherwise is quite difficult chairside.

Aim

The aim of this study is to assess the association between dental caries status, risk assessment, and oral health-related quality of life (OHRQoL) in 3-6-year-old children, before and after undergoing treatment under nitrous oxide inhalation sedation.

Materials and methods

Eighty children were assessed for caries risk and dental status using the caries risk assessment for treatment (CRAFT) tool. Nitrous oxide sedation was employed for dental treatment, and assessments were conducted pre-treatment and post-treatment. Statistical analyses performed were paired t-tests, correlation analyses, and Chi-Square tests.

Results

The study revealed a mean age of 4.033 ± 0.96, with a balanced gender distribution (47.5% boys, 52.5% girls). Post-treatment, significant negative correlations were observed between OHRQoL and treatment outcomes, emphasizing improved oral health-related quality of life. Associations were found between OHRQoL, pufa index, and caries risk post-treatment.

Conclusion

The current research suggests that treatment done under nitrous oxide sedation could improve the OHRQoL in preschool children. Integrating risk assessment and OHRQoL enhances understanding, facilitating targeted prevention and treatment strategies. Addressing untreated dental caries is imperative for optimizing the overall well-being of children in this age group.

## Introduction

In early childhood, dental caries prevention is paramount [1]. Timely assessments conducted by both dental professionals and parents play a pivotal role in caries prevention. Factors contributing to caries risk in children encompass diverse elements such as inadequate oral hygiene practices, limited fluoride availability, excessive sugar intake, intermittent snacking, and parental oversight [2]. Consequently, the assessment of dental status alongside caries risk evaluation emerges as a crucial precursor to formulating effective treatment strategies [3]. Within the age range of 3-6 years, children often exhibit heightened anxiety and a limited capacity to comprehend intricate dental procedures [4]. For these young patients, nitrous oxide sedation emerges as a viable, cost-effective, and non-invasive alternative to the typically invasive behavior management techniques or general anesthesia, which may not yield desirable outcomes due to their inability to grasp instructions, often resulting in distress [5].

This study centers on evaluating children within this age group for caries risk and dental status (before and after treatment) under nitrous oxide sedation. The selection criteria focused on children affected by Early Childhood Caries (ECC), denoting the presence of decayed, missing (due to caries), or filled tooth surfaces in primary teeth. ECC, prevalent in about 50% of the population, extends its impacts beyond dental tissues [6]. Its repercussions encompass a spectrum ranging from dietary constraints, compromised nutrition, disrupted sleep, school absenteeism, hindered academic focus, diminished self-esteem, impaired social interactions, hindrances in speech development, and significantly impacting overall growth and cognitive development [7]. The outcomes of untreated ECC extend beyond the child. It exerts stress on familial finances, manifesting in sleep disruptions for caregivers and loss of work hours. This accentuates the imperative of addressing untreated dental caries, as it notably debilitates the quality of life.

Caries risk assessment (CRA) is a pivotal tool for predicting future caries and helps in guiding evidence-based clinical decisions [8]. In this pursuit, the caries risk assessment for treatment (CRAFT) tool emerges as an indigenous, interview-based tool developed for ascertaining caries risk in children that primarily focuses on dietary habits, fluoride exposure, decay, and related factors reported by parents [9].

Traditional treatment paradigms, predominantly influenced by normative needs, often diverge from perceived needs [10]. Addressing the triggers for seeking treatment, notably pain and sleep disruptions, is critical. Moreover, the focus on normative needs lacks synchronization with the patient's actual needs, thereby hindering the effectiveness of dental care. A shift toward a holistic perspective encompassing health and psychological well-being stands indispensable in evaluating dental needs. Assessment of dental needs ensures optimal treatment outcomes. This reframing advocates for prioritizing improvements in quality of life by enhancing functioning and psychosocial well-being as the primary goals of dental care [11].

The conceptualization of the Oral Health-Related Quality of Life (OHRQoL) indicator emerges as a visionary step, recognizing the significant impact of oral health on daily activities and overall well-being [11]. Incorporating OHRQoL assessments into needs assessment offers a comprehensive understanding of oral health challenges. This facilitates the design of targeted prevention and treatment strategies. Despite its paramount significance, OHRQoL has not been systematically integrated into needs assessment [10]. The Early Childhood Oral Health Impact Scale (ECOHIS), developed to gauge the impact of oral health in children from a familial standpoint, remains underutilized in comprehensive assessments in terms of a socio-dental approach [12]. Treating children under six years old can be challenging due to their limited understanding and fear of the unknown. Nitrous oxide sedation serves as an effective treatment strategy to alleviate anxiety and facilitate successful treatment completion. It serves as an alternative to invasive behavior management techniques like hand-over-mouth exercise and pharmacological methods such as general anesthesia. This paper endeavors to bridge these gaps, integrating risk assessment and OHRQoL to assess the intricate associations between dental caries status, risk assessment, and OHRQoL in 3 to 6-year-old children before and after full mouth rehabilitation using nitrous oxide inhalation sedation.

## Materials and methods

Study design

The single-arm clinical trial was carried out from April 2023 to October 2023, in the Department of Pediatric and Preventive Dentistry following the approval of the Institutional Review Board. The sample size of 80 was determined based on an initial pilot study carried out within the pediatric department. Informed consent was obtained from the parents of the children who were willing to participate in the study.

Inclusion criteria

Children aged between 3 and 6 years, children with a minimum of one dental caries (requiring pulpectomy) in each arch, according to dmft scoring criteria, children demonstrating Frankl behavior ratings of 2 or 3, denoting negative and positive behavior during dental procedures, and children classified under ASA 1 (American Society of Anesthesiologists), indicating normal healthy patients without systemic diseases, were included.

Exclusion criteria

Children who have undergone any dental sedation other than nitrous oxide conscious sedation, children with a history of previous dental treatment under general anesthesia, children with special healthcare needs or cognitive impairments that will affect their ability to understand or respond during dental examinations and treatment, children exhibiting habitual mouth breathing patterns, children with a history of severe dental trauma or fractures in the primary dentition, and children with any congenital or acquired craniofacial anomalies affecting the dental arches were excluded.

Pre-operative assessment

In this study, young children accompanied by their guardians were evaluated before treatment initiation. The initial phase involved a comprehensive oral examination conducted by the operator. Parents actively participated by filling out the Early Childhood Oral Health Impact Scale (ECOHIS), offering invaluable insights into the OHRQoL experienced by their children. This questionnaire comprises six domains which can be rated on a quantifiable scale of never, occasionally, or often [13]. The question proforma for OHRQoL is given in Table 1.

**Table 1:** Early Childhood Oral Health Impact Scale (ECOHIS) questionnaire [13]

Questions	Scoring		
Child symptoms domain: How often has your child had pain in teeth, mouth or jaw?	Never	Occasionally	Often
Child function domain: How often has your child…. had difficulty in breathing? had difficulty eating food? had difficulty in pronouncing words? missed pre-school or daycare or school?			
Child psychological domain: Had trouble sleeping? Been irritated or frustrated?			
Child self-images/social interaction domain: Avoided smiling or laughing due to bad breath/pain/discolored/broken teeth? Avoided talking?			
Parent distress domain: How often have you been upset about dental problems/ treatment? How often have you been guilty of dental problems/ treatment?			
Family functions domain : How often have you or your family taken time off work? How often has your child had dental problems or treatment had a financial impact on the family?			

Caries risk assessment was also expertly appraised by the same operator using the CRAFT app-a digital tool, categorizing risks as low, moderate, or high focusing on dietary habits, fluoride exposure, and decay-related facets. The questionnaire for caries risk assessment is given in Table 2.

**Table 2:** Questionnaire from the CRAFT digital tool for caries risk assessment [9] CRAFT: Caries risk assessment for treatment

S.No	Question	Yes/No
Diet		
1	More than 2 between meal exposures per day of items with sugar, jaggery or Honey (chocolates, soft drinks, sweets, cakes, etc.,) Starch (chips, wafers)	
2	Milk bottle	
3	Syrup medicines or sweet pills (long term - more than a month)	
Fluoride		
1	Brushing with fluoride-containing toothpaste	
2	Receiving six monthly fluoride applications from a dentist	
3	Using fluoride mouth rinse	
4	Living in an area with high fluoride in the water	
Decay		
1	Does your child have untreated cavities?	
2	Did he or she have cavities treated in the past?	
3	Do you (mother/father) have untreated cavities	
Others		
1	Does your child frequently catch infections or has conditions suggestive of low immunity (cold/cough/stomach upset/asthma, etc.,)	
2	Does your child have White chalky or brown patches on the teeth?	
3	Does your child frequently get a dry mouth?	
4	Does your child have crowded teeth?	

This tool's value lies in its ability to offer personalized guidance, aiding dentists in tailoring preventive strategies and treatment plans according to the identified risk levels. The recommendations derived from CRAFT scores serve as practical directives for patients and caregivers. It empowers them with targeted oral health guidance to mitigate potential risks identified during the assessment. This personalized approach fosters a more proactive involvement from both parties, ultimately contributing to more effective preventive measures and treatment decisions. For instance, CRAFT advises against using fluoride mouth rinse in children under 6 years old. However, in this study, participants aged 3 to 6 were surveyed about their use of fluoride mouth rinse. If they reported using it, the dentist would provide counseling to their parents on this matter. The CRAFT recommendations for patients are given in Table 3.

**Table 3:** Caries risk assessment for treatment (CRAFT) tool recommendations for patients [9]

Category	Recommendation
Diet	Reduce sugar exposure to less than two exposures per day preferably at meals
Fluoride	Consult your doctor for a topical fluoride application to prevent tooth decay. Use a fluoride mouth rinse once a day (for > 6 years).
Decay	Start the treatments for the decayed teeth of the child. Take necessary precautions not to transmit decay bacteria from parent to child (by avoiding sharing spoons or kissing particularly if parents have decayed teeth). Start treatments for the decayed teeth of the parents.
Other	Consult your family doctor/pediatrician regarding recurrent illness, allergies, etc., Strengthen teeth enamel with use of remineralizing agents (consult your dentist for the same)

The CRAFT recommendations for dentists are given in Table 4.

**Table 4:** Caries risk assessment for treatment tool (CRAFT) recommendations for dentists [9]

Risk category	Preventive measures	Restorative/surgical measures	Follow-up
Very low/No risk	Home measures -patient counseling related to Dietary advice for sugar reduction and bottle feeding station age-appropriate toothbrushing (with fluoride toothpaste). Fluoride mouth rinse and other remineralizing agents as per the need.	None	Six months
Low risk	Home measures - patient counseling related to dietary advice for sugar reduction and bottle feeding cessation. Age-appropriate tooth brushing (with fluoride toothpaste). Fluoride mouth rinse and other remineralizing agents as per the need. Office measures consider minimally invasive approaches; watchful observation for arrested lesions Fluoride varnish application Fissure sealants Silver diamine fluoride	Consider minimally invasive approaches surface modification selective caries removal and restoration with RMGIC Fissure sealant Preventive resin restorations Smart restorations other intracoronal and extracoronal restorations or other treatments only if required	Six months
Moderate risk	Home measures- patient counselling related to Dietary advice for sugar reduction and bottle feeding cessation Age-appropriate toothbrushing (with fluoride toothpaste). Fluoride mouth rinse and other remineralizing agents as per the need. Office measures- consider Minimally invasive approaches watchful observation for arrested lesions Fluoride varnish application Fissure sealants Silver diamine fluoride	Consider minimally invasive as well as restorative/ surgical measures surface modification selective caries removal and restoration with RMGIC Fissure sealant Preventive resin restorations Smart restorations other intracoronal and extracoronal restorations Pulp therapy Extractions	Three -Six months
High risk	Home measures- patient counselling related to Dietary advice for sugar reduction and bottle feeding cessation Age-appropriate toothbrushing (with fluoride toothpaste). Fluoride mouth rinse and other remineralizing agents as per the need. Office measures- consider Minimally invasive approaches watchful observation for arrested lesions Fluoride varnish application Fissure sealants Silver diamine fluoride	Consider minimally invasive as well as restorative/ surgical measures surface modification selective caries removal and restoration with RMGIC Fissure sealant Preventive resin restorations Smart restorations other intracoronal and extracoronal restorations Pulp therapy Extractions	Three months or more frequently

Dental indices, including pufa (pulpal involvement, ulceration, fistula, abscess) and dmft (decay, missing, filled teeth), were recorded during the examination by the operator to evaluate the dental status [14]. The dental indices used for assessing dental status are given in Table 5.

**Table 5:** Dental indices for dental status assessment [14] dmft-decay, missing, filled teeth ; pufa - pulpal involvement , ulceration, fistula, abscess

Scoring criteria				
Decay (d)	Missing (m)	Filled (f)	Total dmft	
* 0-1 less severity ; 2-4 moderate severity; >5 high severity				
Pulpal involvement (p)	Ulceration (u)	Fistula (f)	Abscess (a)	Total pufa
* 0 no severity; 1-3 mild severity ; 4-6 moderate severity; >7 high severity				

All the parameters analyzed in this study, OHRQoL, caries risk, and dental status, were evaluated using indices and applications sourced from existing literature evidence [9,15,16].

Clinical procedure

Following the rigorous pre-operative assessment phase, the children underwent dental treatment across four appointment sessions (one quadrant per visit) under the influence of nitrous oxide conscious sedation by a single operator. The operator was pre-trained in treating children under conscious sedation. During each appointment, comprehensive treatment for that particular visit was completed. Initially, oxygen was administered for about five minutes after which titration was started. The dose of conscious sedation in the current study was maintained at 30% nitrous oxide-70% oxygen throughout the procedure. The sedation was reversed after completion of the pulpectomy procedure. After the end of the procedure, oxygen was administered for five minutes. The patient was observed for 1 hour before discharge. 

Post-operative assessment

Subsequently, after a six-month interval post-final treatment, a detailed reassessment ensued. Parents once again filled out the ECOHIS scale, while the same operator reevaluated the pufa, dmft indices, and caries risk using the CRAFT app. The study's meticulous design aimed to decipher the impact of full mouth rehabilitation under nitrous oxide sedation on diverse dental parameters like dental status, caries risk, and OHRQoL. A comprehensive understanding of the dental implications on children's oral health and quality of life was observed.

Statistical analysis

Statistical analyses were conducted using IBM SPSS Statistics for Windows, Version 23 (Released 2015; IBM Corp., Armonk, New York, United States), with the significance level established at p<0.05.The association between the parameters and their correlation were statistically analyzed by the Chi-square test and Spearman correlation test respectively. 

## Results

The pre-treatment and post-treatment mean total age of the children included in the trial were 3.91 ± 0.679 and 3.94 ± 0.700 respectively. Out of which, 47.5% were boys and 52.5% were girls. There was no statistically significant difference (p=0.2776) between the girls and boys included, which implies that there was a nearly equal distribution of the population. There was no statistically significant difference in the age group before and after treatment, as none of the participants outgrew the age limit included in the trial (p=0.159). The age-related subgroup analysis of the participants included in the trial can be found in Table 6.

**Table 6:** Age-related subgroup analysis of the participants *p<0.05 statistically significant

	Age n (%)	
	Pre-treatment	Post-treatment
3 years	22 (27.5%)	22 (27.5%)
4 years	43 (53.8%)	41 (51.2%)
5 years	15 (18.8%)	17 (21.3%)
6 years	0	0
p Value	0.159	

The evaluation of pre-treatment assessment and post-treatment outcomes following nitrous oxide sedation was analyzed using a paired t-test. There was a statistically significant difference in the OHRQoL, pufa and caries risk assessment scores before and after treatment (p=0.000). The dmft scores did not show any change before and after treatment as the decayed teeth were counted as filled or missing due to extraction, post-treatment.Upon testing correlation, a negative correlation (-0.098) was evident in the OHRQoL assessment, indicating a potential inverse relationship between oral health-related quality of life and treatment outcomes. This implies that as the treatment outcomes improve, the reported score for OHRQoL decreases, suggesting an enhancement in the overall oral health-related quality of life. There was a strong, positive correlation seen in the pufa and caries risk assessment scores before and after treatment. The results can be found in Table 7.

**Table 7:** Correlation between OHRQoL, dental status, and caries risk assessment before and after nitrous oxide inhalation sedation OHRQoL: Oral health-related quality of life; dmft: decayed, missing, filled teeth; pufa: pulpal involvement, ulceration, fistula, abscess; *p<0.05 statistically significant; paired t-test and Spearman correlation test are the tests of significance

Variable	Mean ± Standard deviation		p-Value	Correlation Coefficient
	First Visit	After treatment completion		
OHRQoL assessment	31.40 ± 6.409	3.53 ± 1.140	0.000*	-0.098
dmft	7.95 ± 0.987	7.95 ± 0.987	1.000	1.000
pufa	6.22 ± 1.276	1.15 ± 0.423	0.000*	0.157
Caries risk assessment	3.44 ± 0.499	1.12 ± 0.324	0.000*	0.048

Upon statistical analysis, there was a significant association after completion of treatment, between OHRQoL - pufa and pufa - caries risk (p<0.05). The results can be found in Table 8.

**Table 8:** Association between OHRQoL, dental status, and caries risk before and after nitrous oxide conscious sedation OHRQoL: Oral health-related quality of life; dmft: decayed, missing, filled teeth; pufa: pulpal involvement, ulceration, fistula, abscess; *p<0.05 statistically significant

Association of variables	First visit		After treatment completion	
	Pearson chi-square value	p-value	Pearson chi-square value	p-value
dmft - OHRQoL	78.799	0.453	20.494	0.154
dmft - pufa	17.286	0.139	12.612	0.050
dmft-caries risk	6.378	0.095	3.705	0.295
OHRQoL - caries risk	22.057	0.686	6.075	0.299
OHRQoL -pufa	103.759	0.488	59.768	0.000*
pufa - caries risk	4.568	0.335	14.053	0.001*

## Discussion

In pediatric dentistry, the mouth and teeth are vital, not only for functions like eating and speaking but also for the psychosocial well-being of children [17]. When children experience tooth loss, it can profoundly impact various facets of their daily lives, including their academic pursuits and social interactions [18].

Beyond the physical and aesthetic consequences, the psychosocial impact is noteworthy. The absence of teeth can affect a child by diminishing their confidence and subjecting them to ridicule from peers and family members [18]. The presence of teeth significantly impacts speech articulation, pronunciation, and the effective digestion of food following thorough chewing. Tooth loss can lead to speech impediments such as lisps or whistling sounds, as well as unhealthy oral habits like tongue thrusting [17]. The social dynamics within the preschool environment may pose challenges as children without proper dental aesthetics may become targets for teasing or mockery [18]. This social aspect, in turn, can contribute to a decline in their self-esteem and overall confidence, highlighting the broader implications of dental issues on a child's holistic well-being. It is crucial to recognize the interconnection of oral health with a child's academic and social experiences [19]. Addressing dental concerns in pediatric patients goes beyond mere restoration. It involves promoting an environment, that nurtures not only their physical health but also their emotional and social development.

ECC is characterized by decayed, missing (due to caries), or filled tooth surfaces in the primary teeth of children under the age of six years. It not only affects dental health but also extends its influence to broader aspects of overall well-being [19]. ECC extends beyond dental tissues, underscoring the urgency for a comprehensive evaluation by affecting dietary habits, nutrition, sleep patterns, academic performance, and psychosocial well-being [19]. Untreated ECC might contribute to adverse cognitive outcomes in children, but the precise mechanisms remain incompletely understood. Possible pathways include compromised mastication affecting the hippocampus. Chronic inflammation induced by the generation of pro-inflammatory cytokines through ECC causes brain damage, sleep disturbances influencing cognitive development, and disruptions to the gut microbiome impacting the gut-brain axis [20]. Furthermore, ECC may result in nutritional deficiencies, elevating the risk to brain development [21]. Implementing early childhood health initiatives to address ECC, is essential for minimizing potential long-term consequences and fostering optimal brain development in young children.

Biria et al. highlight the impact of dental caries on salivary Interleukin-8 (IL-8) levels in children, emphasizing its role in the immune response to caries [22]. Neutrophils in saliva combat bacterial infections, aided by processes like chemotaxis, opsonization, and phagocytosis [22]. IL-8, a chemotactic factor for neutrophils, enhances lysozyme secretion against bacteria by stimulating neutrophil accumulation [22].

Caries risk assessment involves evaluating various factors contributing to the likelihood of future caries development. Factors including dietary habits, fluoride exposure, and decay-related facets are assessed using the CRAFT digital tool. Understanding the association between caries risk assessment and dental status is crucial in devising effective preventive and treatment strategies [23]. One of the primary motivations behind this study lies in the utilization of nitrous oxide sedation as an alternative to the more commonly employed general anesthesia in dental procedures for young children, specifically in the 3 to 6-year age range [24]. This age range presents particular challenges due to children's limited ability to cooperate and comprehend complex dental procedures, often resulting in high levels of anxiety and non-compliance [25]. Traditionally, dental treatments for this age group necessitate general anesthesia due to the challenges in managing their behavior and ensuring procedural compliance [25]. However, the reliance on general anesthesia entails inherent risks, including the need for hospitalization, potential side effects, and a more invasive approach, which can be distressing for both children and their caregivers. In contrast, this study stands out by focusing on the application of nitrous oxide sedation, a less invasive, non-invasive, and conscious sedation method that allows for chairside dental procedures [26]. Nitrous oxide offers a safer alternative, reducing the need for hospitalization and mitigating potential risks associated with general anesthesia [27]. The utilization of the conscious sedation method not only addresses the challenges posed by behavioral management but also emphasizes a more patient-centric approach. This enhances the overall comfort and safety of the pediatric dental experience [27]. The findings of this study reflect statistically significant alterations in OHRQoL, dental indices pufa (pulpal involvement, ulceration, fistula, abscess), dmft (decay, missing, filled teeth), and caries risk assessment post-nitrous oxide sedation. It emphasizes the pivotal role of effective dental interventions in augmenting the overall well-being of children within this age cohort.

Previously, the concept of Quality of Life (QoL) lacked clarity and substance. The evolving socio-dental approach integrates conventional measures with OHRQoL tools prioritizing QoL enhancement. The Early Childhood Oral Health Impact Scale (ECOHIS) emerges as a sensitive, validated, and reliable tool, particularly effective when assessing children's oral health [15]. Our findings resonate with prior research, showcasing a direct correlation between ECOHIS scores and dental caries severity. As supported by a previous study, a higher caries burden consistently corresponds to elevated ECOHIS scores, underlining the detrimental impact of dental caries on QoL [28].

The statistical analysis of the present study revealed a significant association between risk assessment outcomes and ECOHIS scores. Notably, children categorized as "high" risk exhibited notably higher ECOHIS scores, underscoring the pronounced effect of increased risk on a child's OHRQoL. The approach to managing dental caries demands a shift towards a risk-oriented strategy, with elevation of quality of life as its central goal. In the current study, at the end of a six-month re-evaluation, all the participants remained within the age group included in the trial of 3 to 6 years. Thus bias related to age-influenced changes in behavior can be ruled out.

Predictive risk assessment, when capable of anticipating OHRQoL, emerges as a powerful motivator for parental and child engagement in preventive initiatives. The correlation between CRAFT and ECOHIS scores solidifies the predictive capability of risk assessment. This underscores the potential to prioritize treatment for children with socio-dental needs, considering both their risk assessment and OHRQoL. A tailored approach based on these assessments can significantly shape targeted interventions, optimizing oral health outcomes while nurturing a positive quality of life for pediatric patients.

The following limitations of the present study when overcome, could pave the way for future research. Extending this study across multiple centers with a larger participant pool would strengthen the credibility of these findings. Attributing the sole improvement in QoL to nitrous oxide sedation might be presumptive. It’s plausible that QoL enhancement could result from any treatment modality. Comparing the impact on QoL between procedures performed using different methods, such as general anesthesia requiring prolonged pre-procedural investigations versus nitrous oxide sedation characterized by intervals between visits, could offer valuable insights. The absence of a control group is indeed a major shortcoming of the current study. Examining the treatment outcomes across various dosages of nitrous oxide would provide added information to existing literature. Evaluating and contrasting the nuanced relationship and discrepancies in QoL outcomes derived from these distinct procedural approaches would significantly contribute to a more comprehensive understanding of pediatric dental care.

## Conclusions

This study substantiates an association between dental caries status and its discernible impact on the OHRQoL in 3 to 6-year-old children. A significant correlation emerged between caries risk when evaluated using the CRAFT tool and its influence on the OHRQoL within the included population. The current research suggests that treatment done under nitrous oxide sedation could improve the OHRQoL in preschool children.
